# Polypharmacy as maintenance treatment in bipolar illness: A systematic review

**DOI:** 10.1111/acps.13312

**Published:** 2021-05-25

**Authors:** Andrea Amerio, Daniel Russo, Norberto Miletto, Andrea Aguglia, Alessandra Costanza, Beatrice Benatti, Anna Odone, Sergio A. Barroilhet, Vlasios Brakoulias, Bernardo Dell’Osso, Gianluca Serafini, Mario Amore, S. Nassir Ghaemi

**Affiliations:** ^1^ Department of Neuroscience, Rehabilitation, Ophthalmology, Genetics, Maternal and Child Health (DINOGMI) Section of Psychiatry University of Genoa Genoa Italy; ^2^ IRCCS Ospedale Policlinico San Martino Genoa Italy; ^3^ Department of Psychiatry Tufts University Boston MA USA; ^4^ Department of Mental Health A.S.L. CN1 Cuneo Italy; ^5^ Department of Psychiatry Faculty of Medicine University of Geneva (UNIGE) Geneva Switzerland; ^6^ Department of Psychiatry ASO Santi Antonio e Biagio e Cesare Arrigo Hospital Alessandria Italy; ^7^ Department of Biomedical and Clinical Sciences Luigi Sacco Luigi Sacco Hospital University of Milan Milan Italy; ^8^ Department of Public Health, Experimental and Forensic Medicine University of Pavia Pavia Italy; ^9^ Center for Quantitative Health Massachusetts General Hospital Boston MA USA; ^10^ Department of Psychiatry Harvard Medical School Boston MA USA; ^11^ Department of Psychiatry Clinical Hospital University of Chile Santiago Chile; ^12^ Western Sydney Local Health District Mental Health Service and School of Medicine Western Sydney University Blacktown Hospital Sydney NSW Australia; ^13^ “Aldo Ravelli” Center for Nanotechnology and Neurostimulation University of Milan Milan Italy; ^14^ Department of Psychiatry and Behavioral Sciences Stanford University Stanford CA USA

**Keywords:** bipolar illness, polypharmacy, maintenance treatment

## Abstract

**Objectives:**

Polypharmacy is common in maintenance treatment of bipolar illness, but proof of greater efficacy compared to monotherapy is assumed rather than well known. We systematically reviewed the evidence from the literature to provide recommendations for clinical management and future research.

**Method:**

A systematic review was conducted on the use of polypharmacy in bipolar prophylaxis. Relevant papers published in English through 31 December 2019 were identified searching the electronic databases MEDLINE, Embase, PsycINFO, and the Cochrane Library.

**Results:**

Twelve studies matched inclusion criteria, including 10 randomized controlled trials (RCTs). The best drug combination in prevention is represented by lithium + valproic acid which showed a significant effect on time to mood relapses (HR = 0.57) compared to valproic acid monotherapy, especially for manic episodes (HR = 0.51). The effect was significant in terms of time to new drug treatment (HR = 0.51) and time to hospitalization (HR = 0.57). A significant reduction in the frequency of mood relapses was also reported for lithium + valproic acid vs. lithium monotherapy (RR=0.12); however, the trial had a small sample size. Lamotrigine + valproic acid reported significant efficacy in prevention of depressive episodes compared to lamotrigine alone.

**Conclusions:**

The literature to support a generally greater efficacy with polypharmacy in bipolar illness is scant and heterogeneous. Within that limited evidence base, the best drug combination in bipolar prevention is represented by lithium + valproic acid for manic, but not depressive episodes. Clinical practice should focus more on adequate monotherapy before considering polypharmacy.

**Summations**

- Polypharmacy is common in maintenance treatment of bipolar illness, but proof of greater efficacy compared to monotherapy is assumed rather than well known.
- Within that limited evidence base, the best drug combination in bipolar prevention is represented by lithium + valproic acid for manic, but not depressive episodes.
- Clinical practice should focus more on adequate monotherapy before considering polypharmacy.

**Limitations**

- The heterogeneity of selected studies and the low certainty of the outcomes represent the main limitations of this review.
- The majority of the studies addressed the addition of a second drug to the maintenance monotherapy in case of failure.
- To date, the field of polypharmacy in bipolar illness maintenance treatment lacks longitudinal RCT in which patients who failed drug A respond better to A+B than to B alone.

In diseases of the mind … it is an art of no little importance to administer medicines properly; but, it is an art of much greater importance and more difficult acquisition to know when to suspend or altogether to omit them

Philippe Pinel. Treatise on insanity. Birmingham, 1806

## 1 INTRODUCTION

Bipolar illness is a disabling mental illness with a high risk of relapse and recurrence affecting about 45 million people worldwide.^1^ In most patients, it is a chronic condition, associated with clinical, psychosocial, and cognitive decline.^2^

Sustained remission of mood episodes has been associated with better outcomes on cognitive performances and brain morphology, while affective recurrences have been shown to have an impact in terms of treatment resistance and disability.^3^, ^4^ Therefore, an effective maintenance treatment is needed to prevent relapses, hence minimize illness progression, reduce residual symptoms, restore functioning and quality of life.

Lithium, some antiepileptics, and second‐generation antipsychotics are recommended and widely used as maintenance treatments.^5^ Commonly it is stated that bipolar illness tends to require polypharmacy in most patients.^5^ Experts and clinicians feel comfortable with this approach, assuming that this disease is too severe to respond to monotherapy in most cases.^6^

In line with the Canadian Network for Mood and Anxiety Treatments (CANMAT) and International Society for Bipolar Disorders (ISBD) 2018 guidelines for the management of bipolar illness, “if therapy with one or a combination of the first‐line agents at optimal doses is inadequate or not tolerated, the next step is to switch to or add on an alternate first‐line agent.”^5^

Despite its wide use in clinical practice, evidence on polypharmacy in bipolar prophylaxis is still scant. Using the possible combinations of couples of first‐line maintenance drugs reported by the CANMAT and ISBD 2018 guidelines, we systematically reviewed the evidence from the literature on bipolar illness maintenance polypharmacy to provide recommendations for clinical management and future research.

## 2 METHODS

This review was conducted according to methods recommended by the Cochrane Collaboration and the Preferred Reporting Items for Systematic Reviews and Meta‐Analyses (PRISMA) guidelines.^7^, ^8^

### 2.1 Information sources and search strategy

Studies were identified searching the electronic databases MEDLINE, Embase, PsycINFO, and the Cochrane Library. We combined the search strategy of free text terms and exploded MESH headings for the topic of bipolar illness maintenance polypharmacy, using the possible combinations of couples of first‐line maintenance drugs as suggested by the CANMAT and ISBD 2018 guidelines for the management of bipolar illness as following: (((((((((((((bipolar disorder) OR (bipolar)) OR (mania) OR (manic)) OR (bipolar depression)) OR (manic‐depressive)) OR (affective psychosis, bipolar[MeSH Terms])) OR (bipolar disorder[MeSH Terms])) OR (bipolar depression[MeSH Terms])) OR (mania[MeSH Terms])) OR (manias[MeSH Terms])) OR (manic depressive psychoses[MeSH Terms])) AND ((((((((((((prevention) OR (maintenance)) OR (prophylaxis)) OR (long‐term)) OR (continuation)) OR (stabilization)) OR (stability)) OR combination)) OR (bipolar disorder/prevention and control[MeSH Terms])) OR (longitudinal studies[MeSH Terms])) OR (prospective studies[MeSH Terms])) OR (retrospective studies[MeSH Terms])) AND ((((((((((((((((lithium AND valproate) OR (lithium AND divalproex)) OR (lithium AND valproic)) OR (lithium AND lamotrigine)) OR (lithium AND asenapine)) OR (valproate AND lamotrigine)) OR (valproic AND lamotrigine)) OR (divalproex AND lamotrigine)) OR (valproate AND asenapine)) OR (valproic AND asenapine)) OR (divalproex AND asenapine)) OR (lamotrigine AND asenapine)) OR (lamotrigine AND quetiapine)) OR (asenapine AND quetiapine)) OR (aripiprazole AND quetiapine)) OR (asenapine AND aripiprazole)) NOT (Review[Publication Type]) NOT (Systematic Review[Publication Type]) NOT (Practice Guideline[Publication Type]) AND ((humans[Filter]) AND (english[Filter]) AND (adolescent[Filter] OR alladult[Filter])). Evidence on combinations of Lithium or Valproate + Aripiprazole and Lithium or Valproate + Quetiapine was not reviewed as this was already assessed within the CANMAT and ISBD 2018 guidelines. The strategy was first developed in MEDLINE and then adapted for use in the other databases (Appendix A). Studies published in English through 31 December 2019 were included. In addition, further studies were retrieved from reference listing of relevant articles and consultation with experts in the field.

### 2.2 Inclusion criteria

#### 2.2.1 Study population and study design

We considered studies that included bipolar adolescents (from 13 to 18 years) and adults (older than 18 years) treated with combinations of couples of first‐line maintenance drugs as suggested by the CANMAT and ISBD 2018 guidelines for the management of bipolar illness. Participants of both sexes were considered. All studies included subjects with a diagnosis of bipolar illness. Given the aim of this review, subjects had to be evaluated after the remission of an acute episode or, if remission was not an explicit inclusion criterion, the study had to include outcomes related to prophylaxis (frequency of relapse/hospitalization, time to relapse/hospitalization).

No exclusion criteria were set for the recruitment setting or for the source of the clinical data. All experimental and observational study designs were included apart from case reports and case series. Narrative and systematic reviews, letters to the editor, and book chapters were excluded.

#### 2.2.2 Comparisons and outcomes

Only studies with two or more treatment groups were included to allow comparison of interventions. Studies were excluded if they provided aggregated efficacy data regarding different drug combinations and the considered polypharmacy was not a combination of CANMAT and ISBD first‐line maintenance treatment options.

The primary outcome was the effectiveness of combinations of first‐line bipolar illness maintenance drugs in terms of clinical relapses or hospitalizations. Secondary outcomes were (I) frequencies of response and remission, (II) time to discontinuation, (III) safety and tolerability of drug combinations.

### 2.3 Study selection and data extraction

Identified studies were independently reviewed for eligibility by two authors (DR and NM) in a two‐step process. A first screening was performed based on title and abstract, and then full texts were retrieved for a second screening. At both stages disagreements by reviewers were resolved by consensus. Data were extracted by two authors (DR and NM) and supervised by a third author (AAm) using an *ad hoc* developed data extraction spreadsheet. The selected studies are presented on a spreadsheet piloted on ten randomly chosen papers and modified accordingly.

Efficacy data were presented at the level of single outcome on a modified version of the Evidence profile template obtained from the Grade Development Tool. Outcomes were ordered in different tables according to the intervention and comparison. All the relevant outcomes (see section “Comparisons and Outcomes”) presented in the papers are reported in this review. Wherever possible, outcomes were reported with effect sizes (ES), confidence intervals (CI), and *p*‐values.

### 2.4 Quality assessments

The quality of individual outcome results was assessed according to the Grading of Recommendations Assessment, Development and Evaluation (GRADE) method with the following parameters: bias, inconsistency, indirectness, imprecision, publication bias, effect size, influence of confounding factors, dose response gradient.^9^ Each outcome had a qualitative grade of certainty assigned from the four possible levels, ranging from very low to high. Outcomes of randomized controlled trials (RCTs) were rated high, and those from observational studies began the grading with moderate level of certainty. Afterward, outcomes were downgraded, respectively, by one or two levels for every parameter showing serious or very serious concerns. We generally followed the criteria from the GRADE handbook to downgrade the outcome certainty. An exception was imprecision which was downgraded because of small number of participants: by 1 level if the study had less than 30 participants per treatment arm, by 2 levels if the study had less than 10 participants per arm, in line with the thresholds used to evaluate the quality of evidence within the CANMAT and ISBD 2018 guidelines. We also downgraded for imprecision by 1 level if the CI of the ES included 0.75 and 1.25 and by 2 levels if it included 0.5 and 1.5, and if the study had lower power for the outcome than estimated in its power analyses (when the result was not significant).

## 3 RESULTS

**FIGURE 1:**
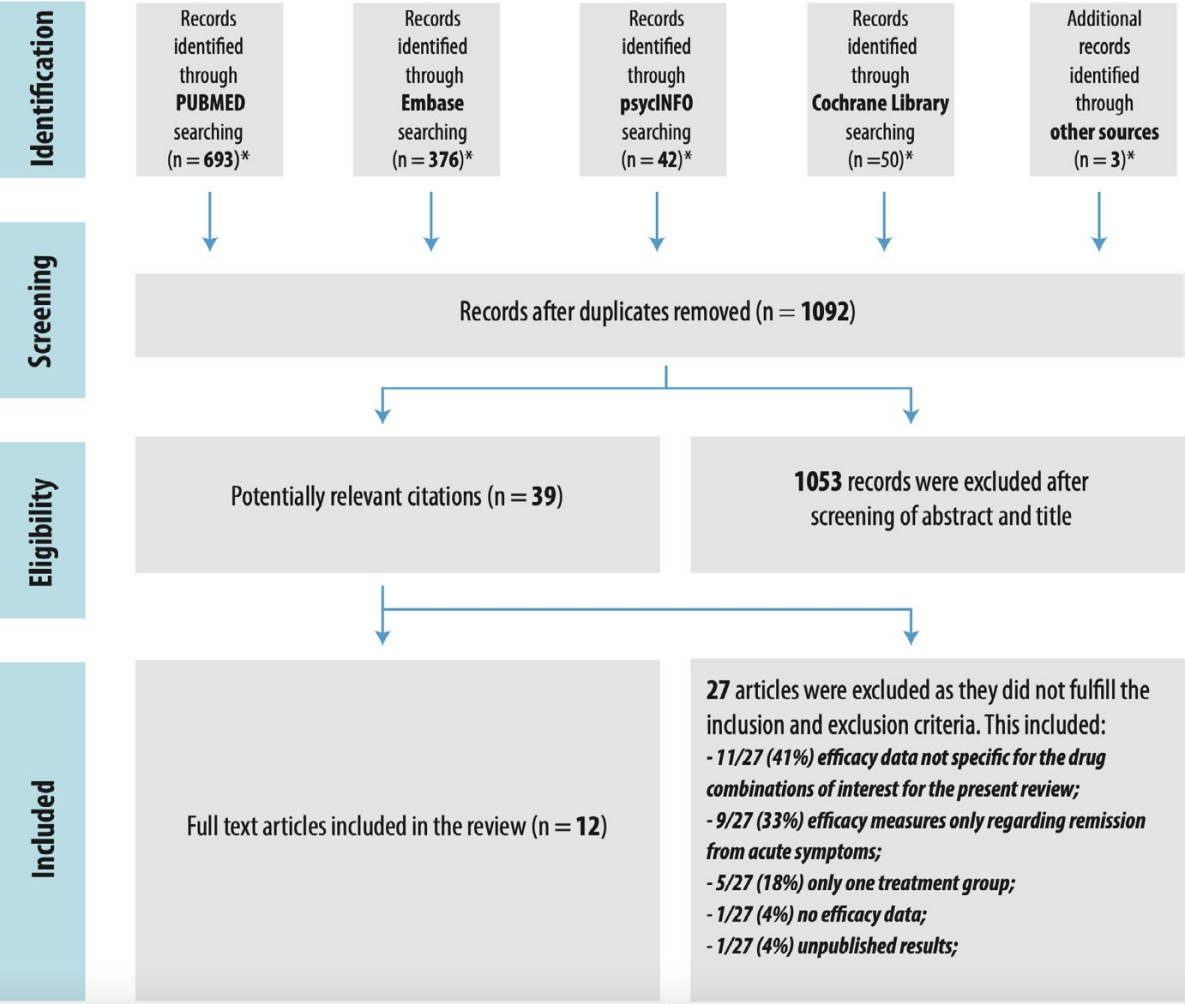
Flow diagram of papers selected. * Search strategy limited to December 2019, English language, human subjects older than 13 years old

The literature search in the four databases yielded 1161 results, and 3 studies were retrieved outside the electronic search through citations in relevant literature in the field. One thousand and ninety‐two studies remained after the removal of duplicates. One thousand and fifty‐three results were excluded on the basis of title and abstract. The remaining 39 items were read in full text, and 27 of them were excluded. The total number of findings from the single databases and the reasons for exclusion of full texts are displayed in Figure 1.

### 3.1 Included studies

Twelve studies matched the inclusion criteria for the present review (Table 1). Six out of ten possible combinations of CANMAT and ISBD 2018 first‐line medications had at least one efficacy study as maintenance treatment.

**TABLE 1:** Studies that met inclusion criteria for systematic review

References	Study design	Country	Study population	N at entry/Retained	Diagnostic assessment	Outcomes
Ahearn EP et al., 2013^11^	RCT	USA	BD (*n* = 1306; mean age = NA)	1306 veterans (1151 M, 155 F)	DSM	Suicide attempts
The BALANCE investigators and collaborators, 2010^13^	Open, randomized study with three active groups	England, Northern Ireland, Scotland, France, USA, Italy	BD (*n* = 459; mean age = 13)	459 screened, 330 randomized in equal numbers between the 3 treatment groups. 263 completed the trial, 167 of these were on treatment, no significant differences in drop‐outs between groups.	DSM‐IV	Primary outcome: time to intervention for an emerging mood episode or hospitalization. Secondary outcomes: time to new intervention for an emerging depressive or manic episode (including mixed and cycling); according to GAF; DSH, EQ‐5D, adverse events; withdrawal from study treatment; adherence to study treatment.
Bowden CL et al., 2012^19^	RCT	USA	BD (*n* = 164; Mean age = 12.4)	164 in the open phase, 86 enrolled in maintenance phase, 45 LTG + PCB, 41 LT G+ VPA	DSM‐IV; MINI	Primary outcome: tolerability and during maintenance phase efficacy on depressive outcome. Severity of the illness and psychopathological features were measured by: MRS, MADRS, CGI‐S and GAS. Subjects were administered the BISS at the point of randomization, week 8 of the maintenance phase, and at study termination.
Carlson BX et al., 2012^21^	RCT	USA, Puerto Rico	BD (*n* = 787; mean age = 39.0 years)	787 enrolled, 352 randomized: 173 LTG + PCB, 178 LTG + ARI; 118 completed	DSM‐IV‐TR;	Hospitalization for a manic or mixed episode; a serious adverse event (SAE) or worsening disease during the study; YMRS >14; MADRS ≥16 for a relapse to a manic episode; a YMRS total score >14 and a MADRS total score >16 for a relapse to a mixed episode; and a YMRS total score ≥14 and a MADRS total score >16 for a relapse to a depressive episode. Secondary outcomes: time to any relapse (manic, mixed, depressive), time to depressive relapse, and time to discontinuation for any reason. Other outcomes: change from baseline in YMRS and MADRS total scores, CGI‐BP, Severity of Illness score (mania, depression, overall), and mean CGI‐BP change from preceding phase score (mania, depression, overall).
Geddes JR et al., 2016^22^	RCT	USA	BD (*n* = 202); Mean age 38.7 years (SD = 11.1)	202 randomized (55 QUE + TLG + PCB, 46 QUE + PCB + Folic acid, 46 QUE + LTG + Folic acid, 55 QUE + PCB + PCB), 103 completed.	DSM‐IV	Primary outcome: improvement in depressive symptoms at 12 weeks with QIDS‐SR16. Manic symptoms were assessed with ASRM. Quality of life was measured at baseline and at 12 weeks and 52 weeks with EQ‐5D‐3L. Data for symptoms and quality of life were provided by participants by the True Colours system via text message, email, or paper.
Kemp DE et al., 2009^14^	RCT	USA	BD‐I (*n* = 149; Age ≥16 and ≤65 years)	149 enrolled, 31 randomized (16 Li + DV, 15 Li + PCB). 42% dropped out before randomization because of poor adherence, 25% poor response, 10% adverse effects	DSM‐IV	Primary Outcome: time to treatment for emerging mood symptoms. Secondary outcomes: study discontinuation for any reason, time to depressive relapse, and time to manic/hypomanic/mixed relapse. A Cox proportional hazards model was used to evaluate differences for the following predictors of outcome: treatment arm assignment, bipolar subtype, index episode at study entry, and substance use disorder diagnosis. A repeated measures mixed‐effects model was used to analyze mean changes in symptom severity scores.
Maina G et al., 2008^18^	Single‐blind, randomized, pilot	Italy	BD‐I or BD‐II (*n* = 56; Age ≥18 and ≤70 years)	56 enrolled, 49 randomized (26 Olanzapine) (23 Lamotrigine) 40 completed 12‐week trial.	SCID‐I; DSM‐IV	Primary outcome: HAM‐A score from to baseline to endpoint. Secondary outcomes: the severity of illness from baseline to endpoint CGI‐S, GAF scales.
Missio G et al., 2019^15^	RCT	Brazil	BD (*n* = 64; mean age 27.8 years)	64 enrolled, randomized 36 Li/VPA, 28 Li/CBZ. Females (66.6%)	SCID‐I	Primary Outcomes: number of participants achieving/maintaining response and remission during the acute and maintenance phases of BD treatment, respectively. Response Acute phase: (symptom severity reduction of at least 50% with no worsening of symptoms in the opposite pole), no response (symptom severity reduction of less than 50%), and loss of response (response followed by symptom severity reduction of less than 50% in at least one visit or at the physician's discretion). Maintenance phase, Remission: symptom severity reduction of at least 75% with no worsening of symptoms in the opposite pole. Secondary outcome: number of participants achieving BD remission during the maintenance phase of treatment.
Solomon DA et al., 1997^16^	RCT	USA	BD (*n* = 12, mania; mean age = 39.6 years)	12 enrolled, randomized 5 Li + VPA, 4 females.	SCID; DSM‐III‐R	Outcomes: recurrences and relapses, side effects of moderate. severe effects for more than 4 weeks, adjunctive medications for at least 4 weeks. Assessment instrument: PSR score of LIFE interview. Patients entered the study during an acute episode (PSR 5 or 6). Partial remission was an improvement in symptoms not meeting the DSM‐III criteria (PSR 3 or 4), patients in recovery had no symptoms for 8 weeks consecutively (PSR 1 or 2). Relapse was defined as a return of symptoms meeting DSM criteria (PSR 5 or 6) during a partial remission, recurrence was a return of symptoms occurring during a remission (PSR 1 or 2).
Szegedi A et al., 2012^20^	RCT	Australia, Czech Republic, India, South Korea, Russia, Taiwan, Thailand, USA	BD (*n* = 326; mean age = 15.9)	326 randomized, 42.0% female, 158 Asenapine; 166 PCB; 116 completed 12 weeks of treatment. (mean age 38.9 years), 44.16% female. 77 enrolled in the extension and 34 completed 52 weeks of treatment.	YMRS; DSM‐IV	Primary outcome: change in YMRS from baseline to week 3, at week 12, response (decrease in YMRS ≥ 50%), remission (YMRS ≤ 12), changes CGI‐BP mania scores. Secondary end points: change from baseline at weeks 3, 12, and other time points on the MADRS total score, CGI‐BP score for severity of depression, and the modified ISST. Secondary outcome: change from baseline at weeks 3, 12, and other time points on (HAM‐A), (CNS), (RDQ), (SF‐36), (Q‐LES‐Q). Extension study outcomes: change from core study baseline in YMRS and MADRS total scores at week 52 and earlier time points. The YMRS response and remission rate at week 52 and change from core study baseline to week 52 in CGI‐BP, HAM‐A, ISST, CNS Vital Signs, SF‐36, RDQ, and Q‐LES‐Q.
Van der Loos MLM et al., 2011^17^	RCT	Netherlands, Spain	BD (*n* = 124: mean age = 11.9)	124 enrolled, randomized 64 Li/LTG, 60 LI/PCB. Non responders to additional LTG or PCB (*n* = 37) (SD = 11.9). Females 67 (54.0%)	DSM‐IV; MADRS; MINI‐Plus	Primary outcome: the relapse or recurrence of a manic or depressive episode defined as a score ≥4 (at least moderate symptoms) on the CGI‐BP severity of depression or mania scale. Secondary outcome: how long the MADRS score was maintained <50% of its baseline value after patients reached this level for the first time during phase 1 or 2.
Wingard L et al., 2019^12^	Prospective cohort	Sweden	BD (*n* = 5713; Mean age not reported 18–75 years)	5713 (*n* = 3261 female) hospitalizations for mania, representing 3772 patients. Treatment failure 4871 cases (85.3%). Monotherapies: Li (*n* = 1 133), VPA (*n* = 525), QUE (*n* = 468), ARI (*n* = 146); Combination therapies: Li+VPA (*n* = 217), Li+QUE (*n* = 314), Li+ARI (*n* = 92), VPA+QUE (*n* = 171), VA+ARI (*n* = 50), Li + VPA + QUE (*n* = 68)	ICD‐10	Primary outcome: treatment failure defined as: (1) discontinuation of medication, (2) switch of medication, or (3) being readmitted to inpatient psychiatric care during an active treatment period.

AED, Antiepileptics Drug; ARI, Aripiprazole; ASRM, Altman Self‐Rating Mania Scale; BD, Bipolar disorder; BISS, Body Image State Scale; BPRS, Brief Psychiatric Rating Scale; BRMS, Beck Rafaelson's Mania Rating Scale; CBZ, carbamazepine; CGI‐BD, Clinical Global Impressions Improvement Scale for Bipolar Disorder; CGI‐BP‐IM, Clinical Global Impressions for Bipolar Disorder Improvement‐Mania; CGI‐BP‐IM, Clinical Global Impressions for Bipolar Illness‐Improvement Mania Scale; CGI‐I, Clinical Global Impression‐Improvement Scale; CGI‐S, Clinical Global Impression‐Severity Scale; CNS, Central Nervous Systems; DSH, Deliberate Self‐Harm; DSM, Diagnostic and Statistical Manual of Mental Disorders; EQ5D, EuroQol; FU, Followed‐up; GAF, Global Assessment of Functioning; GAS, Global Assessment Scale; HAM‐A, Hamilton Anxiety Scale; HAM‐D, Hamilton Depression Rating Scale; HAM‐D, Hamilton Depression Scale; HRSD‐25, Hamilton Rating Scale for Depression; ICD, International Classification of Diseases; ISST, Trial Scale for Suicidal Thinking; Li+, lithium carbonate; LTG, Lamotrigine; MADRS, Montgomery–Åsberg Depression Rating Scale; MINI, Mini‐International Neuropsychiatric Interview; NA, Not Available; NIMH, National Institute of Mental Health prospective Life Chart Methodology; PCB, Placebo; PSR, Psychiatric Status Rating; QIDS‐SR16, Quick Inventory of Depressive Symptomatology; Q‐LES‐Q, Quality of Life Enjoyment and Satisfaction Questionnaire; QUE, Quetiapine; RCT, Randomized controlled trial; RDQ, Readiness to Discharge Questionnaire; SAE, Serious Adverse Event; SCID, Structural Clinical Interview for DSM; SF‐36, Short Form‐36; UKU, Udvalg for Kliniske Undersøgelser; VPA, valproate; YMRS, Young Mania Rating Scale.

No efficacy literature was found for the lamotrigine + asenapine combination and for treatments with 2 antipsychotics (asenapine + aripiprazole, asenapine + quetiapine, aripiprazole + quetiapine). One RCT protocol for the aripiprazole + quetiapine combination has been published, but results were not available on the date of our search.^10^

Two observational studies with multiple treatment groups,^11^, ^12^ 4 RCT on lithium + valproic acid,^13^, ^14^, ^15^, ^16^ 2 RCTs on lamotrigine + lithium,^17^, ^18^ 1 RCT on lamotrigine + valproic acid,^19^ 1 RCT on asenapine + lithium or valproic acid,^20^ 1 RCT on aripiprazole + lamotrigine,^21^ and 1 RCT on quetiapine + lamotrigine,^22^ were included.

Eight RCTs presented control groups treated with one of the drugs in the combination^14^, ^15^, ^16^, ^17^, ^19^, ^20^, ^21^, ^22^; 7 of them had also placebo treatment in the monotherapy group.^14^, ^16^, ^17^, ^19^, ^20^, ^21^, ^22^

Two RCT had active treatment control groups with another combination therapy.^15^, ^18^

### 3.2 Outcomes

#### 3.2.1 Combination of lithium and valproic acid

Only the BALANCE (Bipolar Affective disorder: Lithium Anticonvulsant Evaluation) study compared the preventive efficacy of valproic acid + lithium vs. valproic acid on mood episodes (Table 2), showing a significant effect on time to mood relapses (HR = 0.57), especially for the manic ones (HR = 0.51).^13^ The effect was significant in terms of time to new drug treatment (HR = 0.51) and time to hospitalization (HR = 0.57).

**TABLE 2:** Combination of lithium and valproic acid

References	№ of patients		Certainty assessment								Effect			
	Combination	Comparison treatment	Study design	Outcome/Event	Follow‐up time	Risk of bias	Inconsistency	Indirectness	Imprecision	Other considerations	Relative (95% CI)	Absolute (95% CI)	Test (*p*‐value)	Certainty
	Valproic acid + Lithium	Valproic acid												
The BALANCE investigators and collaborators, 2010^13^	110 –	110 69.0%	Randomized trials	Time to intervention for any mood episode	24 months	Serious^a^	Not serious	Not serious	Not serious	None	**HR 0.57** (0.40–0.80)	**−20%** (−32 to −8)	Log‐rank (0.0014)	⨁⨁⨁◯ MODERATE
The BALANCE investigators and collaborators, 2010^13^	110 –	110 45.0%	Randomized trials	Time to intervention for depressive episode	24 months	Serious^a^	Not serious	Not serious	Serious^b^	None	**HR 0.70** (0.46–1.07)	**−11%** (−21 to +2)	Log‐rank (0.11)	⨁⨁◯◯ LOW
The BALANCE investigators and collaborators, 2010^13^	110 –	110 45.0%	Randomized trials	Time to intervention for manic episode	24 months	Serious^a^	Not serious	Not serious	Not serious	None	**HR 0.51** (0.32–0.80)	**−19%** (−28 to −7)	Log‐rank (0.003)	⨁⨁⨁◯ MODERATE
The BALANCE investigators and collaborators, 2010^13^	110 –	110 68.0%	Randomized trials	Time to new drug treatment	24 months	Serious^a^	Not serious	Not serious	Not serious	None	**HR 0.57** (0.40–0.80)	**−20%** (−31 to −8)	Log‐rank (0.0014)	⨁⨁⨁◯ MODERATE
The BALANCE investigators and collaborators, 2010 ^13^	110 –	110 23.0%	Randomized trials	Time to hospital admission	24 months	Serious^a^	Not serious	Not serious	Not serious	None	**HR 0.51** (0.27–0.96)	**−11%** (−16 to −1)	Log‐rank (0.038)	⨁⨁⨁◯ MODERATE
The BALANCE investigators and collaborators, 2010^13^	110 –	110 44.0%	Randomized trials	Time to medication discontinuation	24 months	Serious^a^	Not serious	Not serious	Very serious^c^	None	**HR 1.01** (0.67–1.53)	**0%** (−12 to +15)	Log‐rank (0.95)	⨁◯◯◯ VERY LOW
Ahearn EP et al., 2013^11^	145	821	Observational studies	Suicide attempts 10,000 months on drug regimen	6 years, mean 19.8 months	Very serious^c^, ^d^	Not serious	Serious^e^	Not serious	None	Valproate + Lithium: 6.3; Valproate: 7.0.		**‐**	⨁◯◯◯ VERY LOW
	Valproic acid + Lithium	Lithium												
The BALANCE investigators and collaborators, 2010^13^	110 –	110 59.0%	Randomized trials	Time to intervention for any mood episode	24 months	Serious^a^	Not serious	Not serious	Serious^b^	None	**HR 0.80** (0.57–1.15)	**−6%** (−18 to +6)	Log‐rank (0.23)	⨁⨁◯◯ LOW
Kemp DE et al., 2009^14^	16 –	15 55.0%	Randomized trials	Time to intervention for any mood episode	6 months	Serious^d^	Not serious	Serious^f^	Very serious ^b^, ^c^, ^g^	None	**HR 0.72** (0.32–1.65)	**−11%** (−32 to +18)	Log‐rank (0.44)	⨁◯◯◯ VERY LOW
Wingard L et al., 2019^12^	217 –	1133 87.0%	Non‐randomized studies	Time to treatment failure	365 days	Very serious^h^	Not serious	Not serious	Not serious	None	**HR 0.72** (0.62–0.85)	**−10%** (−15 to −5)	‐	⨁⨁◯◯ LOW
The BALANCE investigators and collaborators, 2010^13^	110 –	110 32.0%	Randomized trials	Time to intervention for depressive episode	24 months	Serious^a^	Not serious	Not serious	Very serious^b^, ^c^	None	**HR 1.06** (0.67–1.67)	**+2%** (−9 to +15)	Log‐rank (0.81)	⨁◯◯◯ VERY LOW
The BALANCE investigators and collaborators, 2010^13^	110 –	110 36.0%	Randomized trials	Time to intervention for manic episode	24 months	Serious^a^	Not serious	Not serious	Serious^b^	None	**HR 0.66** (0.41–1.07)	**−11%** (−15 to +2)	Log‐rank (0.09)	⨁⨁◯◯ LOW
The BALANCE investigators and collaborators, 2010^13^	110 –	110 20.0%	Randomized trials	Time to hospital admission	24 months	Serious^a^	Not serious	Not serious	Very serious^b^, ^c^	None	**HR 0.72** (0.38–1.38)	**−5%** (−12 to +7)	Log‐rank (0.32)	⨁◯◯◯ VERY LOW
Kemp DE et al., 2009^14^	217 –	1133 20.4%	Non‐randomized studies	Time to hospital admission	365 days	Very serious^h^	Not serious	Not serious	Not serious	None	**HR 1.05** (0.78–1.40)	**−1%** (−41 to +69)	‐	⨁⨁◯◯ LOW
The BALANCE investigators and collaborators, 2010^13^	110 –	110 45.0%	Randomized trials	Time to medication discontinuation	24 months	Serious^a^	Not serious	Not serious	Very serious^b^, ^c^	None	**HR 0.89** (0.60–1.34)	**−4%** (−15 to +10)	Log‐rank (0.58)	⨁◯◯◯ VERY LOW
Kemp DE et al., 2009^14^	217 –	1133 20.2%	Non‐randomized studies	Time to medication discontinuation	365 days	Very serious^e^	Not serious	Not serious	Not serious	None	**HR 0.63** (0.44–0.89)	**−7%** (−11 to −2)	‐	⨁⨁◯◯ LOW
The BALANCE investigators and collaborators, 2010^13^	110 –	110 58.0%	Randomized trials	Time to new drug treatment	24 months	Serious^a^	Not serious	Not serious	Serious^b^	None	**HR 0.80** (0.56–1.14)	**−8%** (−20 to +5)	Log‐rank (0.21)	⨁⨁◯◯ LOW
Kemp DE et al., 2009^14^	217 –	1133 46.5%	Non‐randomized studies	Time to new drug treatment	365 days	Very serious^h^	Not serious	Not serious	Not serious	None	**HR 0.62** (0.49–0.79)	**−14%** (−20 to −8)	‐	⨁⨁◯◯ LOW
Solomon DA et al., 1997^16^	0/5 (0.0%)	5/7 (71.4%)	Randomized trials	Relapse frequency	12 months	Not serious	Not serious	Serious^i^	Very serious ^c^, ^g^	None	**RR 0.12** (0.01–1.79)	**−63%** (−71 to +56)	Chi‐squared (0.014)	⨁◯◯◯ VERY LOW
Ahearn EP et al., 2013^11^	145	436	Observational studies	Suicide attempts 10,000 months on drug regimen	6 years, mean 19.8 months	Very serious^c^,^d^	Not serious	Serious^j^	Not serious	None	Valproate + Lithium: 6.3; Lithium: 7.7.		‐	⨁◯◯◯ VERY LOW
	Valproic acid + Lithium	CBZ + Lithium												
Missio G et al., 2019^15^	33	27	Randomized trials	Time to relapse	22 weeks	Very serious^a^, ^d^, ^k^	Not serious	Not serious	Serious^g^	None	Time‐to‐relapse curves were not significantly different between groups		Log‐rank (0.45)	⨁◯◯◯ VERY LOW

CBZ, Carbamazepine; CI, Confidence interval; HR, Hazard Ratio; MD, Mean difference; RR, Risk ratio. Explanations:

^a^ Open‐label trial.

^b^ Low power for the outcome.

^c^ Large CI including significantly reduced and increased effect.

^c^ No measures of disease severity or comorbidities available.

^d^ Incomplete outcome reporting.

^e^ Days of prescription used as proxy of days of treatment.

^f^ Population of rapid cycling patients with recent alcohol / illicit drug abuse or dependence.

^g^ Small sample size.

^h^ Case severity assessed by length of index hospitalization and presence of psychotic symptoms. Lack of concealment for allocation.

^i^ 4 patients never recovered from the index episode, but they were equally distributed between the treatment arms.

^j^ Days of prescription used as proxy of days of treatment.

^k^ No definition of relapse.

Four studies allowed the comparison in efficacy as maintenance treatment of valproic acid + lithium versus lithium (Table 2). The pilot trial conducted by Solomon and colleagues was the only study showing a significant effect on the frequency of mood relapses of the drug combination versus lithium monotherapy (relative risk, RR = 0.12), although several shortcomings as the small sample size (<10 patients per arm).^16^ The other studies, 2 RCTs and 1 observational study,^12^, ^13^, ^14^ consistently suggest some advantages in terms of time to mood relapse or treatment failure in the combination treatment group (hazard ratio, HR, between 0.72 and 0.80), although these results did not reach statistical significance. The BALANCE study also shows a non‐significant delay in time to manic episodes with polypharmacy (HR = 0.66), although the same tendency does not apply to depressive episodes (HR = 1.06).^13^

One observational study measured suicide attempts in populations exposed to valproic acid + lithium (6.3/10,000 months of exposure), lithium monotherapy (7.7/10,000 months of exposure), and valproic acid monotherapy (7/10,000 months of exposure) (Table 2).^11^ The study did not provide any statistical tests to compare these measures.

Finally, one RCT compared valproic acid + lithium vs. carbamazepine + lithium (Table 2). The study states there were no significant differences between the time‐to‐relapse curves (no ES reported, *p* = 0.45).^15^

#### 3.2.2 Combinations of lamotrigine and lithium or valproic acid

One RCT compared lamotrigine + divalproex with lamotrigine alone (Table 3).^19^ The study failed to show statistically significant effect for time to depressive relapse (HR = 0.67) for the combination treatment. However, secondary outcomes such as the number of patients reaching the cutoff for depressive symptoms (RR = 0.66) and patients with discontinuation due to depressive symptoms (RR = 0.30), showed significant efficacy for the drug combination in prevention of depressive episodes. Statistical significance was not reached for the risk of manic relapses (RR = 0.61).

**TABLE 3:** Combinations of lamotrigine and lithium or valproic acid

			Certainty assessment									Effect		
References	Combination	Comparison treatment	Study design	Outcome/Event	Follow‐up time	Risk of bias	Inconsistency	Indirectness	Imprecision	Other considerations	Relative (95% CI)	Absolute (95% CI)	Test (*p*‐value)	Certainty
	Lamotrigine + Valproic acid	Lamotrigine												
Bowden CL et al., 2012^19^	41	45 (66.7%)	Randomized trials	Time to reach cutoff for depressive symptoms	8 months	Not serious	Not serious	Not serious	Very serious^a^, ^b^	None	**HR 0.67** (0.37 to 1.25)	−**15%** (−33 to +8)	Log‐rank (>0.05)	⨁⨁◯◯ LOW
Bowden CL et al., 2012^19^	18/41 (43.9%)	30/45 (66.7%)	Randomized trials	Patients reaching cutoff for depressive symptoms	8 months	Not serious	Not serious	Not serious	Not serious	None	**RR 0.66** (0.44 to 0.99)	**−23%** (−37 to −1)	Chi‐squared (0.03)	⨁⨁⨁⨁ HIGH
Bowden CL et al., 2012^19^	3/41 (7.3%)	11/45 (24.4%)	Randomized trials	Patients with discontinuation due to depressive symptoms	8 months	Not serious	Not serious	Not serious	Not serious	None	**RR 0.30** (0.09 to 1.00)	**−17%** (−22 to 0)	Chi‐squared (0.03)	⨁⨁⨁⨁ HIGH
Bowden CL et al., 2012^19^	5/41 (12.2%)	9/45 (20.0%)	Randomized trials	Patients relapsing into manic episode	8 months	Not serious	Not serious	Not serious	Very serious^b^	None	**RR 0.61** (0.22–1.67)	**−8%** (−16 to +13)	Chi‐squared (0.33)	⨁⨁◯◯ LOW
	Lamotrigine + Lithium	Lithium												
Van der Loos MLM et al., 2011^17^	30	25	Randomized trials	Frequency of depressive relapses	52 weeks	Very serious^c^, ^d^	Not serious	Not serious	Serious^e^	None	These percentages maintained higher levels in the combination treatment group than in the monotherapy group throughout the study		‐	⨁◯◯◯ VERY LOW
Van der Loos MLM et al., 2011^17^	30	25	Randomized trials	Time to relapse into depressive episode	52 weeks	Very serious^c^, ^d^	Not serious	Not serious	Serious^e^	None	The time to relapse or recurrence was longer for the combination treatment group than for the monotherapy group: median time 10.0 months [95% confidence interval (CI): 1.1–18.8] versus 3.5 months (95% CI: 0.7–7.0), respectively.		‐	⨁◯◯◯ VERY LOW
	Lamotrigine +Lithium	Olanzapine +lithium												
Maina G et al., 2008^18^	7/18 (38.9%)	14/22 (63.6%)	Randomized trials	Response frequency	12 weeks	Serious^f^	Not serious	Not serious	Serious^g^	None	**RR 0.61** (0.32–1.18)	**−25%** (−43 to +11)	Pearson 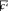 (0.12)	⨁⨁◯◯ LOW
Maina G et al., 2008^18^	5/18 (27.8%)	12/22 (54.5%)	Randomized trials	Remission frequency	12 weeks	Serious^f^	Not serious	Not serious	Serious^g^	None	**RR 0.51** (0.22–1.18)	**−27%** (−43 to +10)	Pearson 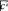 (0.09)	⨁⨁◯◯ LOW

CI, Confidence interval; HR, Hazard Ratio; RR, Risk ratio​. Explanations:

^a^ Low statistical power due to lower number of events than expected.

^b^ Large CI including appreciable benefit and harm.

^c^ Paroxetine added to achieve response, unequally distributed in the treatment arms.

^d^ Effect sizes and p‐values not reported.

^e^ Small sample size in the maintenance phase.

^f^ Researchers not blinded.

^g^ Low number of participants.

Another RCT studied the efficacy of the combination of lamotrigine + lithium vs. lithium (Table 3).^17^ The study recruited patients who were depressed at the baseline and allowed the use of paroxetine to reach remission from the acute episode. Paroxetine was unequally used in the treatment groups (more in the monotherapy group), and results were reported narratively without statistical testing. Study results reported that more patients maintained the responder status in the combination treatment group than in the monotherapy group and the median time to depressive episode was higher with a combination treatment (10 months) compared to monotherapy (3.5 months).

Lamotrigine + lithium has been also compared to olanzapine + lithium combination for the treatment of comorbid anxiety in another RCT (Table 3).^18^ The study reported a significant reduction of anxiety in both groups, although it lacks a control group with placebo or monotherapy. Therefore, a reduction of anxiety cannot be generalized as an effect of the addition of lamotrigine or olanzapine to lithium or vice versa. Moreover, the study did not find a statistically significant difference between the olanzapine and lamotrigine combinations, although frequencies of response and remission of anxiety symptoms were higher with the olanzapine combination compared to the lamotrigine combination (RR = 0.61 for response; RR = 0.51 for remission).

#### 3.2.3 Combinations involving atypical antipsychotics

A single RCT compared aripiprazole + lamotrigine versus lamotrigine (Table 4) without reaching a statistical significance.^21^ However, a trend toward significance was found in time to manic or mixed episode (HR = 0.6) and time to any episode (HR = 0.7), in favor of the combination treatment. Time to depressive episode (HR = 0.8) was not significantly different between the two groups.

**TABLE 4:** Combinations involving atypical antipsychotics

Reference	№ of patients		Certainty assessment								Effect		Test (*p*‐value)	Certainty
	Combination	Comparison treatment	Study design	Outcome/Event	Follow‐up time	Risk of bias	Inconsistency	Indirectness	Imprecision	Other considerations	Relative (95% CI)	Absolute (95% CI)		
	Aripiprazole + Lamotrigine	Lamotrigine												
Carlson BX et al., 2012^21^	178 –	173 23.0%	Randomized trials	Time to manic or mixed relapse	52 weeks	Serious^a^	Not serious	Not serious	Serious^b^	None	**HR 0.6** (0.3 to 1.0)	**−8%** (−15 to 0)	Log‐rank (0.055)	⨁⨁◯◯ LOW
Carlson BX et al., 2012^21^	178 –	173 42.0%	Randomized trials	Time to any relapse	52 weeks	Serious^a^	Not serious	Not serious	Serious^b^	None	**HR 0.7** (0.5 to 1.0)	**−10%** (−18 to 0)	Log‐rank (0.058)	⨁⨁◯◯ LOW
Carlson BX et al., 2012^21^	178 –	173 24.0%	Randomized trials	Time to depressive relapse	52 weeks	Serious^a^	Not serious	Not serious	Very serious^c^	None	**HR 0.8** (0.5 to 1.4)	**−4%** (−11 to +8)	Log‐rank (0.361)	⨁◯◯◯ VERY LOW
	Quetiapine + Lamotrigine	Quetiapine												
Geddes JR et al., 2016^22^	31/101 (30.7%)	39/101 (38.6%)	Randomized trials	New treatments for depression	52 weeks	Not serious	Not serious	Serious^d^	Very serious^c^	None	**RR 0.84** (0.58 to 1.24)	**−6%** (−16 to +9)	– (0.38)	⨁◯◯◯ VERY LOW
Geddes JR et al., 2016^22^	9/101 (8.9%)	12/101 (11.9%)	Randomized trials	New treatments for mania or mixed state	52 weeks	Not serious	Not serious	Not serious	Very serious^b^, ^c^	None	**RR 0.67** (0.29 to 1.56)	**−4%** (−8 to +7)	– (0.35)	⨁⨁◯◯ LOW
	Asenapine + Lithium or Valproic acid	Lithium or Valproic acid												
Szegedi A et al., 2012^20^	41	36	Randomized trials	Time to depressive or manic adverse effect	52 weeks	Serious^e^	Not serious	Serious^f^	Not serious	None	Time to first mood episode showed no statistically significant difference between groups		Log‐rank (0.442)	⨁⨁◯◯ LOW
Szegedi A et al., 2012^20^	9/41 (22.0%)	5/36 (13.9%)	Randomized trials	Frequency of manic or depressive adverse effects	52 weeks	Not serious	Not serious	Serious^f^	Very serious^c^	None	**RR 1.58** (0.58 to 4.29)	**+8%** (−6 to +46)	Chi‐squared (0.361)	⨁◯◯◯ VERY LOW

CI, Confidence interval; HR, Hazard Ratio; RR, Risk ratio. Explanations:

^a^ More patients with rapid cycling BD and mixed episodes within the monotherapy arm.

^b^ Low statistical power due to lower number of events than expected.

^c^ Large CI including significant benefit and harm.

^d^ Not every patient in the FU reached remission from depression, new intervention might also include treatment for the index episode.

^e^ Effect sizes of log‐rank tests not reported.

^f^ Stabilization was not required to enter in the extension study.

Quetiapine + lamotrigine combination was compared to lamotrigine alone in one RCT (Table 4) showing non‐significant reductions in the risks of new treatments for depression (RR = 0.84) and mania (RR = 0.67).^22^

The combination of asenapine + lithium or valproate vs. lithium or valproate was tested in a single RCT (Table 4).^20^ Results narratively reported that time to first mood episode did not show significant differences between the two groups in the 52‐week maintenance phase. Frequencies of manic or depressive symptoms, reported as adverse effects, were higher in the combination treatment group, although not significantly (RR = 1.58).

#### 3.2.4 Safety and tolerability

The combinations of couples of first‐line maintenance drugs suggested by the CANMAT and ISBD 2018 guidelines demonstrated a safe and adequate tolerability profile (Table 5). Serious adverse events and discontinuations due to adverse events were low both in poly‐ and monotherapy treatment groups. No new or unexpected adverse events were reported that differed from the established profile for each individual medication as monotherapy in maintenance treatment. Three out of twelve studies (25%) did not mention side effects in the results.^11^, ^12^, ^19^

**TABLE 5:** Studies that mentioned side effects

References	Side effects
Ahearn EP et al., 2013^11^	NR
The BALANCE investigators and collaborators, 2010^13^	Most participants who responded to (lithium [95%, *N* = 52], valproate [92%, *N* = 48], combination [100%, *N* = 57]) reported at least one non‐serious adverse event during follow‐up.
Bowden CL et al., 2012^19^	NR
Carlson BX et al., 2012^21^	The three most common adverse events were akathisia [10.8%, 6.1% for ARI + LTG and PCB + LTG, respectively; number needed‐ to‐harm (NNH) = 22], insomnia (7.4%, 11.5%), and anxiety (7.4%, 3.6%). Mean weight change was 0.43 kg and 1.81 kg, respectively (last observation carried forward, *p* = 0.001). Rates of ≥7% weight gain with ARI + LTG and PCB + LTG were 11.9% and 3.5%, respectively (NNH = 12).
Geddes JR et al., 2016^22^	No side effects related to CT group are mentioned.
Kemp DE et al., 2009^14^	10% (*N* = 15) discontinued during the open‐label phase because of AE; weight gain (33%), gastrointestinal discomfort (27%), tremors (20%), dizziness (7%), cognitive difficulties (7%), and polyuria/polydipsia (7%) the most common AE; tremors and polyuria/polydipsia, in both treatment groups. A significant increase in alanine transaminase levels occurred in the Li+ and VPA CT group (+19.60 U/L) compared to the LI + monotherapy group (−30.83 U/L; *p *= 0.029).
Maina G et al., 2008^18^	Over 20% of subjects receiving LTG (*N* = 5) experienced an increase in tension and inner unrest; of them, one also experienced an increase in anxiety symptoms and another reported a reduced duration of sleep; 2 of the 3 subjects with an increase in anxiety symptoms also reported a reduced duration of sleep, while 2 of the 4 patients with reduced duration of sleep also reported an increase in anxiety symptoms; 34.8% of patients (*N* = 8) had at least one of the above‐mentioned AEs in the LTG group.
Missio G et al., 2019^15^	Side effects differed significantly between groups only in the first week of treatment (*p* = 0.021), and there were more side effects in the Li/VPA group. Also, the Li/VPA group gained weight (+2.1 kg), whereas the Li/CBZ group presented slight weight loss (−0.2 kg).
Solomon DA et al., 1997^16^	No side effects related to CT group are mentioned.
Szegedi A et al., 2012^20^	5% or more of asenapine patients (sedation, somnolence, depression/depressive symptoms, oral hypoesthesia, and increased weight) in the 12‐week core study. Adjunctive asenapine to lithium or valproate was well tolerated for up to 52 weeks.
Van der Loos MLM et al., 2011^17^	No difference between LTG and PCB in the prevalence of any AE. The total amount of AEs in both groups was remarkably low for a 68‐week follow‐up.
Wingard L et al., 2019^12^	NR

AE, Adverse Event; ARI, Aripiprazole; CBZ, Carbamazepine; CT, Combination Therapy; Li+, Lithium carbonate; LTG, Lamotrigine; NNH, Number needed‐to‐harm; NR, not reported; PCB, Placebo; SAE, Serious Adverse Event; VPA, Valproate.

## 4 DISCUSSION

Studies from the literature on polypharmacy in bipolar illness maintenance treatment are sparse, heterogeneous, and often underpowered, so data interpretation requires caution. However, given the available scientific evidence supporting a statistically significant efficacy in bipolar prevention, some observations can be made (Table 6).

**TABLE 6:** Summary of the specific advantages of combination treatments in maintenance

Combination	Comparison treatment	Manic relapse	Depressive relapse	Inter‐episodic symptoms
Valproic acid + Lithium	Valproic acid	+	+/−	–
Valproic acid + Lithium	Lithium	+/−	–	–
Valproic acid + Lithium	CBZ + Lithium	–	–	–
Lamotrigine + Valproic acid	Lamotrigine	+/−	+	–
Lamotrigine + Lithium	Lithium	–	+/−	–
Lamotrigine + Lithium	Olanzapine + lithium	–	–	+/−^a^
Aripiprazole + Lamotrigine	Lamotrigine	+/−	–	–
Quetiapine + Lamotrigine	Quetiapine	+/−	–	–
Asenapine + Lithium or Valproic acid	Lithium or Valproic acid	–	–	–

+: clear advantage; +/−: possible advantage; –: no advantage/no data/inconclusive data; CBZ, Carbamazepine.

^a^ Advantage given by the comparison treatment.

Findings from BALANCE reported that lithium + valproic acid is the only drug combination showing a statistically significant effect in terms of time to mood relapse compared to valproic acid monotherapy.^13^ More than 40% relative benefit was irrespective of baseline severity of illness, maintained for up to 2 years, and most apparent in prevention of manic relapse. BALANCE was a randomized, open‐label, three‐group trial, designed to imitate routine use of the agents in clinical practice relapse prevention, with up of 24 months of follow‐up. This trial presented no serious methodological issues, except for it being an open‐label study, designed mainly to compare combination therapy with monotherapy, with a relatively small sample (*N* = 110) per treatment arm. On the contrary, BALANCE could neither confirm nor refute a benefit of combination therapy compared with lithium monotherapy. In particular, for depression relapse, lithium monotherapy was as effective as combined therapy, and thus, the addition of valproate was not helpful for added efficacy.

Lithium + valproic acid might also offer some advantages on bipolar relapse or recurrence prevention compared to lithium alone, as reported by a clinical trial conducted in l997.^16^ The study was a randomized pilot trial designed to compare the efficacy of a combination therapy (Lithium + divalproex sodium) with monotherapy (lithium), with up of 12 months of follow‐up. The study had several shortcomings, including the small sample size (less than ten people per arm).

The second drug combination reaching a statistically significant efficacy in bipolar illness maintenance treatment is represented by lamotrigine + divalproex that reduced the risk for depressive episode and for trial discontinuation due to depressive symptoms compared to lamotrigine.^19^ The study was a randomized, double‐blind, parallel group trial designed to compare the efficacy of a combination therapy (lamotrigine + divalproex) with monotherapy (lamotrigine), with up of 8 months of follow‐up. The use of a sample size with inadequate power for a strong test of several hypothesis and the enrollment of an insufficient number of bipolar patients to support separate analyses limited the generalization of the results. It could be also hypothesized that a longer duration of the study could allow the detection of a larger number of manic episodes, hence demonstrating an effect for adjunctive divalproex on manic episodes.

Moreover, evidence from the literature supporting an efficacy in bipolar prevention, although non‐statistically significant, is available for the following drug combinations: lithium + valproic acid compared to lithium,^12^, ^13^, ^14^ lamotrigine + lithium compared to lithium,^17^ valproic acid + lithium compared to carbamazepine + lithium,^15^ aripiprazole or quetiapine + lamotrigine compared to lamotrigine.^21^, ^22^

The impact of the duration of the follow‐up on the observed effect sizes can be complex. While longer duration allows more chances for the outcome events to happen, it might also increase the number of drop‐out events. Interestingly, the studies reaching a statistically significant result had a significant difference in duration, BALANCE lasted 24 months,^13^ while Bowden et al. lasted 8 months^19^; thus, there is not a clear relationship between duration of the follow‐up and the positive results. Nonetheless, some of the selected studies might have shown a positive result with a longer follow‐up, allowing for the detection of more manic episodes.^21^, ^22^

As noted previously, experts often comment that most patients with bipolar illness receive polypharmacy, and they endorse this practice on the grounds of the complexity of response in this condition.^23^ However, the scientific evidence for such practice, as shown here, is still weak, and the belief that polypharmacy is more effective than the best monotherapy needs to be proven by more clinical studies. The complexity of the problem rises further questions about the clinical populations that might benefit from polytherapy and about the specific advantages or disadvantages of each drug combination.

A key caveat to most claims of efficacy of polypharmacy is that they tend to involve designs where patients are preselected to fail a single agent in monotherapy, and then are either continued in the failed monotherapy group or randomized to the addition of a second agent. Since the monotherapy group is already proven to be ineffective, such a design is biased in favor of polypharmacy. Instead, patients should be randomized from the beginning to monotherapy or combined therapy, to test the hypothesis whether two treatments are more effective inherently than one. Very few studies have this design.

If designs are limited to the inclusion to failed monotherapy, then the generalization of results would not involve inherent superiority of polypharmacy to monotherapy, but only added benefit after failed monotherapy. These distinctions are important to make since many clinicians do not provide adequate monotherapy doses and durations of treatment before rapidly moving to polypharmacy practice.

Furthermore, nonadherence seriously limits the effectiveness of any maintenance treatment. Thus, lack of adherence should be always considered in case of treatment failure, especially in the populations at risk, such as people with treatment‐related side effects, comorbid personality, or substance use disorders.^24^

## 5 LIMITATIONS AND STRENGTHS

The main limitations of the review are the heterogeneity of the studies and the low certainty of the outcomes, ranging from very low to moderate in most instances. Quality issues of single outcomes in the studies are detailed in Tables 2, 3, and 4.

Studies with similar outcomes were limited to lithium and valproic acid combinations. A meta‐analysis was not conducted given the paucity of these outcomes, the different study designs, follow‐up times, confounding factors, and inclusion criteria. Therefore, a qualitative review was preferred to address the specific characteristics of the studies. Results are reported as they were available in the original manuscript, and only minor analyses have been done in some instances, such as the calculation of the relative risks and the absolute outcomes with the respective confidence intervals. No stratification of results across different bipolar subtypes was performed.

The main strength of this review is its being systematic and its including the entire scientific evidence published so far on the main medical databases. Moreover, the quality assessment process performed through the GRADE system, allows transparency and systematic assessment of critical issues within selected studies.

## 6 CONCLUSIONS

There is minimal evidence to support the use of combinations of drugs for bipolar illness maintenance treatment. Keeping in mind scantiness and heterogeneity of the available literature, the best drug combination in bipolar prevention is represented by lithium + valproic acid which has been shown to be more effective in preventing manic relapse compared to valproic acid monotherapy, though not for depression relapse, where lithium monotherapy was very effective.

To date, the field of polypharmacy in bipolar illness maintenance treatment lacks longitudinal RCT in which patients who failed drug A respond better to A+B than to B alone. Progress in this area would serve to facilitate more rigorous prophylaxis against manic and depressive relapses. Future research efforts may lead to more grounded guidelines, which are greatly needed in a recurrent and disabling condition as bipolar illness.

## CONFLICT OF INTERESTS

Dr. Amerio, Dr. Russo, Dr. Miletto, Dr. Aguglia, Dr. Costanza, Dr. Benatti, Prof. Odone, Dr. Barroilhet, Prof. Brakoulias, Prof. Dell’Osso, Prof. Serafini, and Prof. Amore report no conflicts of interest. Prof. Ghaemi is employed by Novartis Institutes for Biomedical Research and holds equity in Novartis.

## AUTHOR CONTRIBUTIONS

Authors AAm, AC, BB, AO, and SAB designed the study and wrote the protocol. Studies were identified and independently reviewed for eligibility by two authors (DR and NM) in a two‐step‐based process. Data were extracted by two authors (DR and NM) and supervised by a third author (AAm) using an ad hoc developed data extraction spreadsheet. Authors AAm, DR, NM, and AAg wrote the first draft of the manuscript. AO, VB, BDO, GS, MA, and SNG carefully revised the final version of the manuscript. Our manuscript has been approved by all authors.

### PEER REVIEW

The peer review history for this article is available at https://publons.com/publon/10.1111/acps.13312.

## APPENDIX A Medline search strategy.

**Table:**

Set	MEDLINE
1	Bipolar disorder
2	Bipolar
3	Manic
4	*Bipolar depression*
5	Manic depressive
6	*Affective psychosis, bipolar*
7	*Mania*
8	*Manias*
9	*Manic depressive psychoses*
10	*Bipolar disorder*
11	Sets 1–10 were combined with “OR”
12	*Prevention*
13	Maintenance
14	Prophylaxys
15	Long‐term
16	Continuation
17	Stabilization
18	Stability
19	Combination
20	*Bipolar disorder/prevention and control*
21	*Longitudinal studies*
22	*Prospective studies*
23	*Retrospective studies*
24	Sets 12–23 were combined with “OR”
25	Lithium AND valproate
26	Lithium AND divalproex
27	Lithium AND valproic
28	Lithium AND lamotrigine
29	Lithium AND asenapine
30	Valproate AND lamotrigine
31	Valproic AND lamotrigine
32	Divalproex AND lamotrigine
33	Valproate AND asenapine
34	Valproic AND asenapine
35	Divalproex AND asenapine
36	Lamotrigine AND asenapine
37	Lamotrigine AND quetiapine
38	Asenapine AND quetiapine
39	Aripiprazole AND quetiapine
40	Asenapine AND aripiprazole
41	Sets 25–40 were combined with “OR”
42	Sets 11 and 24 and 41 were combined with “AND”
43	Set 42 was limited to December 31^st^, 2019, Humans, English language, Subjects older than 18 years old

Words written in *italic* were used as MeSH headings, the others were used as free text.
